# Improving health outcomes among older adults in India: effectiveness and implementability of a novel comprehensive geriatric assessment based intervention

**DOI:** 10.12688/wellcomeopenres.19796.2

**Published:** 2023-12-18

**Authors:** Jaya Singh Kshatri, Susan Shenkin, Stewart Mercer, David Weller, Subrata Kumar Palo, Sandipana Pati, Daisy Janssen, Sanghamitra Pati

**Affiliations:** 1 Regional Medical Research Centre, Indian Council of Medical Research, Bhubaneswar, Odisha, 751023, India; 2 Usher Institute, The University of Edinburgh, Edinburgh, Scotland, EH89AG, UK; 3 Centre for Population Health Sciences, The University of Edinburgh, Edinburgh, Scotland, EH89AG, UK; 4 Joint Director, Training, State Institute of Health and Family Welfare, Bhubaneswar, Odisha, 751001, India; 5 CAPHRI School for Public Health and Primary Care, Maastricht University, Maastricht, Minderbroedersberg, 6211LK, The Netherlands

**Keywords:** Comprehensive geriatric assessment, CGA, rural older adults, Implementation science, pilot trial, RCT

## Abstract

**Background:**

There is significant evidence on the benefits of comprehensive assessment in older adults. But this evidence is primarily from western countries and in secondary care settings. National policies in India recognize this need and envision community-based screening and facility-based assessment programs integrated into the care pathways for the elderly. However, this is yet to translate into specific interventions, primarily due to lack of complex interventions necessary and evidence of their effectiveness. This study aims to design and pilot an integrated (Community + Facility) Elderly Health Status Assessment and Screening (EHSAS) intervention to improve health outcomes of older adults and assess its feasibility for implementation in Indian rural settings.

**Methods:**

We propose a hybrid design where we will build the complex intervention, develop and validate the tools needed, pilot it using an exploratory cluster randomized trial and evaluate its implementatbility using the Exploration-Preparation-Implementation-Sustainment (EPIS) framework.

**Conclusions:**

This study will fill critical gaps in evidence regarding the effectiveness of geriatric screening and assessment in community and primary care settings in low-middle income countries and provide validated tools and implementation models for adoption into national programs.

**Registration:**

CTRI/2023/07/055661

## Plain Language Summary

Comprehensive assessment of older adults has proven benefits in hospital settings. However, little is known about the effectiveness and feasibility of such a program in community and primary health care facilities in low- and middle-income countries, such as India. We propose to build such an intervention, test its effectiveness, and demonstrate its feasibility so that this could be integrated into existing national programmes for older adults in India and elsewhere. This study will provide important tools and evidence that in the long run would benefit both the service providers and the intended clients.

## Introduction & rationale

The increasingly aging population in low and middle income countries (LMIC) such as India poses unique social and health challenges ^1^. This disproportionately impacts rural health systems as over two thirds of older adults in India live in rural areas. Odisha, an eastern state, has one of the highest proportions (>86%) of older adults (aged ≥60 years) living in rural areas in India ^2, 3^. This aging population needs community based interventions that include screening and comprehensive assessments aimed at integrated care ^4^. One such intervention is Comprehensive Geriatric Assessment (CGA).

CGA is a multidimensional, multidisciplinary process to identify medical, psycho-social, nutritional, and functional needs of older adults and develop an integrated care plan to address these needs ^5, 6^. Several systematic reviews have shown that CGA is effective in reducing mortality, disability, and morbidity, and improving clinical outcomes in older adults ^5, 7– 13^. However, the evidence on CGA is predominantly from high income countries and targeted at hospitalized older adults ^5, 14^. Research on the effectiveness and strategies for CGA in primary care and community settings is lacking, particularly from low- and middle-income countries ^10^.

In India, the need for community-based screening and CGA has been recognized in its National Policy for Older People and reflected in the National Program for HealthCare of the Elderly (NPHCE). However, the same has not yet been translated into practice or specific interventions ^15– 19^. The primary reason for this evidence-to-practice gap is the conspicuous absence of clearly defined methods for practitioners to perform CGA or implement them into national programs. Further, our recent systematic scoping review revealed there were no multi-dimensional tools that have been validated yet in South Asia, which can be used in such an intervention ^20, 21^.

**Figure 1.**
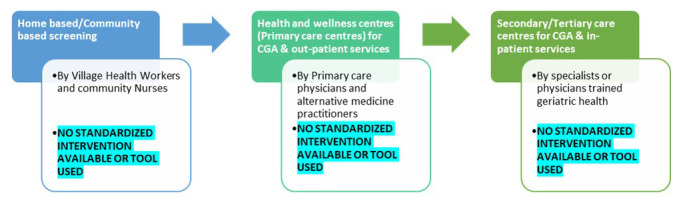
Gaps in the proposed older persons’ care pathway under the National program for Health Care of the Elderly in India (colored boxes) and gaps identified (highlighted).

Figure 1 highlights the gaps in care pathways of older adults in India and Table 1 summarizes the gaps in knowledge identified by previous systematic reviews on the topic and how the proposed study plans to address those gaps.

**Table 1.** Evidence gaps identified and how the study intends to fill those.

Current gaps	How this study will fill those gaps
**Identifying target population**	
**Better methods of identifying the ideal target population** ** for CGA ^5, 12, 23^**	Evidence on effectiveness of community-based screening to identify those in need of a CGA.
**Geriatric Screening and assessment**	
**No locally validated tools to use for CGA ^20, 21^**	Design and validate a set of tools that can be used for CGA in India (and possibly other South Asian countries)
**Limited evidence on role of CGA in LMICs ^5, 7– 14^**	Evidence (from LMIC) on effectiveness of multidisciplinary screening in community and CGA in primary care settings.
**Interventions based on CGA**	
**Lack of standardization in the geriatric screening and** ** assessment processes ^20, 21, 23^**	Standardize the assessment process in the context of the national program (NPHCE) in India
**No CGA-based interventions or evidence on their feasibility/** **effectiveness in primary healthcare settings ^5, 12, 24^**	Build and evaluate the feasibility and effectiveness of a CGA-based intervention in rural community and primary care settings.
**Outcome measures**	
**Limited evidence on the role on Patient Reported Outcome** ** Measures (PROMs) in CGA ^5^**	Evaluate effect of CGA on PROMs (quality of life and self-rated health)
**Implementation designs**	
**Inadequate evidence on feasibility of integrating CGA-** **based interventions into existing programs ^5^**	Evaluate the acceptability and feasibility of the implementation process in context of existing national programs (e.g. NPHCE)

There is a need for an approach to improve health outcomes among older adults in India by developing methods for assessment and implementation, and building capacity in the healthcare of older people.

### Research questions

▪ What is the feasibility and likely benefit of an integrated Elderly Health Status Assessment and Screening (EHSAS) intervention in improving health outcomes among older adults residing in rural areas of Odisha?
▪ What factors could facilitate the future implementability of such an intervention?

### Objectives

a. To design and validate a complex Elderly Health Status Assessment and Screening (EHSAS) intervention for community-based screening and primary healthcare based CGA in older adults aged ≥60 years.
b. To test the feasibility and likely effectiveness of this intervention, by a phase-II exploratory cluster randomized controlled trial (RCT), on the quality of life among adults aged ≥60 years in rural areas of Tigiria, Odisha.
c. To assess the acceptability of the intervention among service providers and clients and identify the factors that could influence its implementation.

### Work that has led up to this protocol

Work at the Model Rural Health Research Unit (MRHRU), operated by the host institute at the proposed study site in Odisha, forms the foundation for this proposal ^22^. A Health and Demographic Surveillance System named the DISHA cohort, that includes approximately 20,000 families from 48 villages, has shown the changing demographic trends of an aging rural population and the subsequent health and social challenges.

This led to the establishment in 2018 of a prospective cohort under the AHSETS study that aims to evaluate the prevalence and interrelationships between multiple domains of health in older adults. The baseline data collection was completed in 2019–2020 for 725 randomly selected rural older adults (aged between 60–106 years, mean = 70 years, 48% females) and we plan to follow them up longitudinally for 2 years ^25– 29^. The AHSETS study has provided evidence of high disease burden (e.g. multimorbidity, disability, frailty, elder abuse) and on the interplay of various physical, psycho-social, and functional domains of health. This has emphasised the need for CGA in rural community settings to identify and manage complex health issues.

The AHSETS study also identified challenges in the assessment and management of older people with complex health needs, in particular the length of time for such detailed assessments and the use of separate and disjointed tools (often with redundant concepts) built for use in a different context. This led to a search for multidimensional and locally relevant health assessment tools. Subsequently, we undertook a systematic scoping review to identify the tools validated for use in South Asian older adults. However, we could not find a single tool that had all the dimensions of CGA ^20, 21, 30^.

## Methods

**Figure 2.**
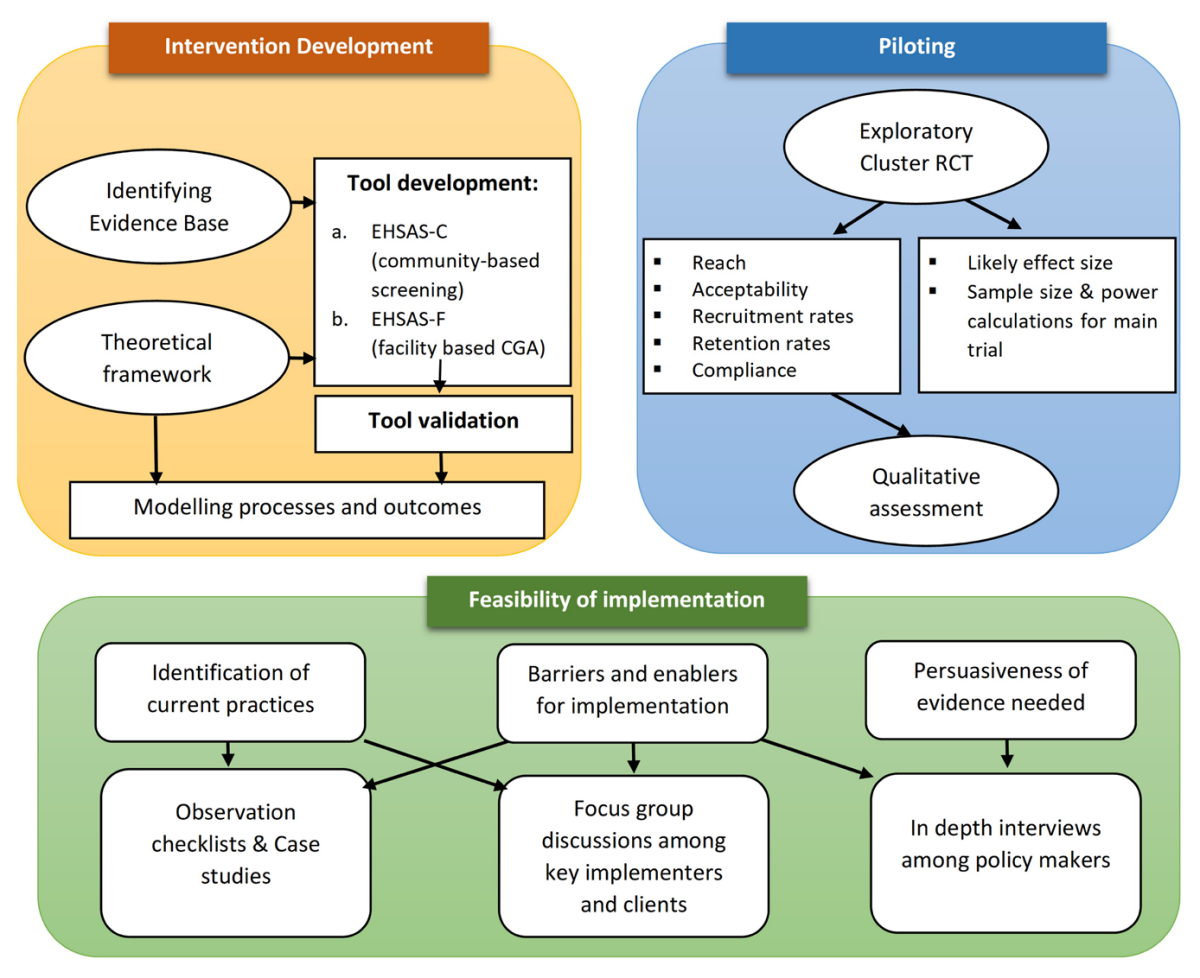
Outline of the proposed study design.

We propose the development and evaluation of a complex CGA-based intervention using the UK Medical Research Council (MRC) framework ^31– 33^. The methodology is presented in three broad steps outlined in Figure 2 and are further elaborated below:

1. Intervention development (EHSAS)
2. Piloting of the intervention
3. Implementation model development and evaluation of feasibility

### Intervention development (EHSAS)

i. Identification of the evidence base
  ▪ *Umbrella review:* We will carry out an umbrella review to synthesize evidence from existing systematic reviews, e.g. 5, 7, 10 focussing on lessons for LMIC and primary care/rural settings. We will follow the guidance provided by the Cochrane Handbook for this umbrella review ^34^.
  ▪ *Scoping review for screening tools:* We have recently completed a review which could not identify any appropriate and comprehensive tools, therefore a new tool needs to be developed and validated for use in the intervention phase ^21^.
  ▪ *DISHA cohort:* We will carry out a descriptive analysis of the socio-demographic and health parameters of older adults collected in the DISHA cohort to describe the characteristics of the target population.
  ▪ *Expert consensus:* The above evidence will be summarized and shared before a panel of experts consisting of geriatricians, public health specialists, primary care physicians, researchers, and psychologists, from both India and UK. Using multiple rounds of consultations, we will attempt to reach a broad consensus on the following characteristics of the intervention package:
    - Components of intervention
    - Parameters of intervention
    - Delivery methods
    - Levels of implementation.

ii. Development and validation of the tools
  ▪ Two tools will be developed and validated:
    ○ EHSAS-C → for community-based multidisciplinary screening
    ○ EHSAS-F → facility based comprehensive assessment (for those identified by screening)
  ▪ The following 9 steps (in 3 phases) outlined by Boateng *et al*. as the best practices in tool development and validation will be followed ( Table 2): ^35^

**Table 2.** The proposed tool development and validation procedures.

Activity		Purpose	Methods
**Phase-I: Item** ** development**	Domain Identification & Item generation	Identify boundaries of the domains and fitting constructs	The domains and constructs relevant for CGA is well established ^5^. We will use deductive methods and existing wider datasets and tools: AHSETS study and the Longitudinal Aging Study of India (LASI) that are available with us to generate preliminary items lists ^29, 37, 38^.
	Content validity	Evaluation of relevance and technical quality of the constructs by experts and representativeness of actual experience from target population	Content validity ratio and Cohens kappa (n= 7-9 domain experts) Face validity (n=10-12 end users)
**Phase-II: Scale** ** development**	Pre-testing	Assess the extent to which questions reflect the domain of interest and answers produce valid measurements	Cognitive interviews (n=10-12 clients in 2 rounds)
	Data collection	Ensure adequate data for scale development and validation	Cross-sectional surveys (n=200, from the AHSETS cohort x 2 rounds, 3 months apart)
	Item reduction	To evaluate item difficulty, discrimination, correlation between items and missing answers	Item difficulty index, inter-item communalities, item-total correlations.
	Factor analysis	Determine the optimal number of factors/domains that fit a set of items	Exploratory factor analysis
**Phase-III: Scale ** **evaluation**	Tests for dimensionality	Identify latent structure of items and their underlying relationships	Confirmatory factor analysis
	Tests for reliability	Assess internal consistency and repeatability	Cronbach’s alpha, test-retest reliability over two time points
	Tests for validity	Establish convergent and discriminant construct validity	Estimate the strength and direction of relationship of constructs with similar tools using multi-trait multi-method matrix

iii. Development of theoretical framework

**Figure 3.**
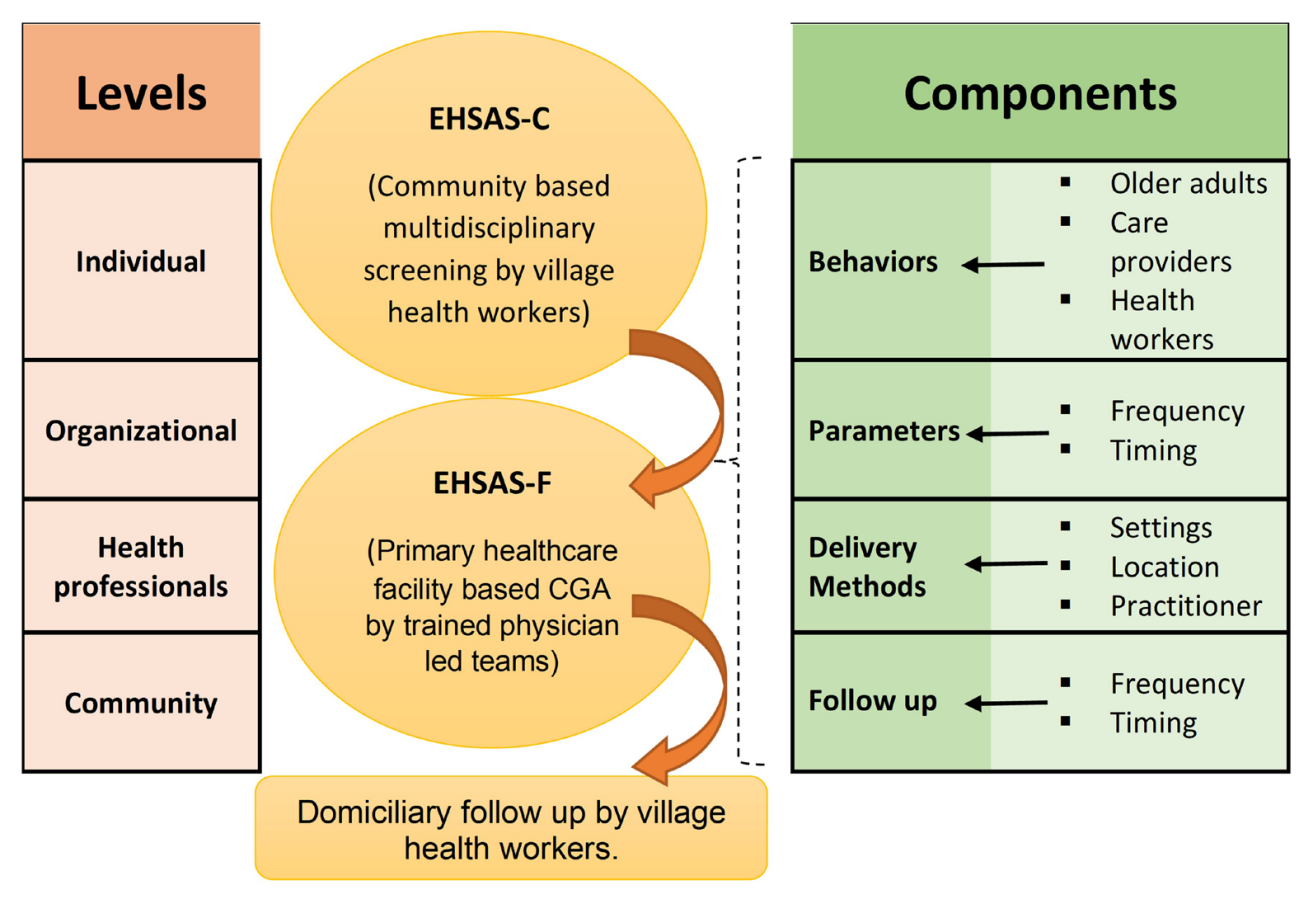
Theoretical framework to develop the EHSAS intervention.

From the existing evidence base, we start with a preliminary theoretical framework (given below in Figure 3) for the possible mechanism of action and efficacy of EHSAS intervention.

To understand the likely processes of change necessary, we will use a qualitative enquiry using key informant in-depth interviews (IDI) (n=20–30) among the key stakeholders (clients at community and facility levels, service providers, policy makers, geriatric support organizations, researchers) using the theoretical domains, component constructs, and eliciting questions for investigating the implementation of evidence-based practice provided by Michie *et al*. ^36^ The findings will be used to further refine and build our theoretical basis for the intervention.

iv. Modelling processes and outcomes

The intervention will be evaluated for its usability, barriers, and enablers. We will aim to understand how the EHSAS intervention will be implemented in community and primary care settings, interrelations between components and levels of the intervention as well as possible pathways of effect on outcomes of interest. Qualitative data will be collected by focus group discussions (FGD) among clients and IDIs among healthcare workers and analysed using the thematic framework in Figure 3.

### Piloting the intervention

**Figure 4.**
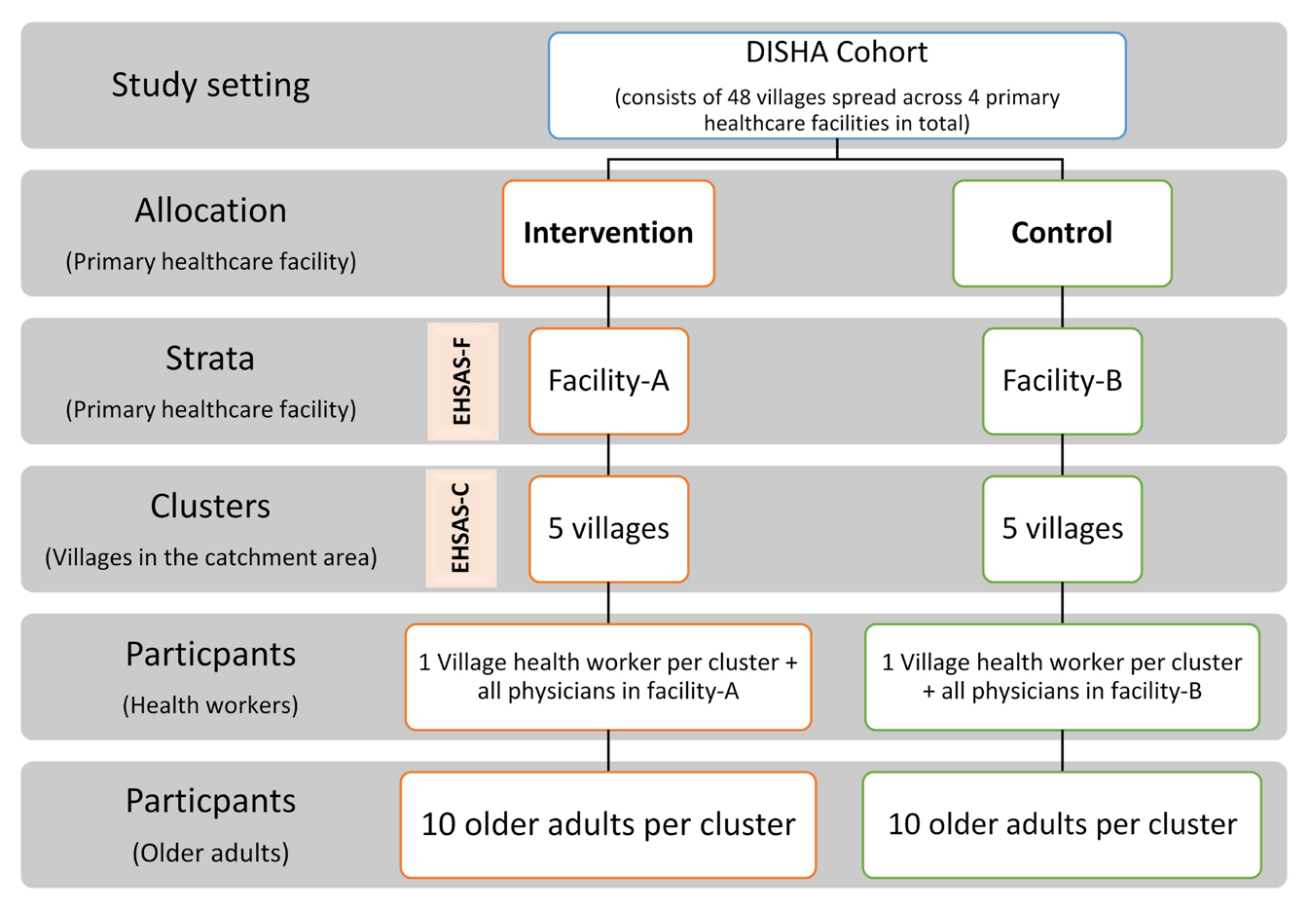
Sampling framework of the cluster randomized trial.

i. Exploratory cluster RCT
  • *Trial design and setting:* A stratified pair-matched exploratory cluster randomized controlled trial will be conducted within villages of the DISHA cohort in Tigiria, Odisha. This corresponds to the UK-MRC phase-II of intervention development guidance ^33^.
  • *Strata and clusters:* Due to the nature of the intervention, a strata of primary health care facilities will be included in the sampling design and clusters will be selected from their catchment areas as shown in Figure 4 below. The clusters (villages) will be matched in pairs based on the demographic characteristics and prevalence of chronic conditions in adults ≥60 years of age in the cluster using the DISHA cohort data.
  • *Selection and allocation of clusters and participants:* The facilities *,* clusters and participants will be selected for the trial using computerised simple random sampling (from the DISHA cohort database) and allocated to intervention/control group by a computer program.
  • *Eligibility criteria, recruitment of participants:* We will include all permanent residents of the selected cluster who are ≥60 years of age. The participants should be able to provide written informed consent and agree to follow up for 24 months. We will exclude those with severe cognitive impairment (as assessed by the Dementia Assessment Rapid Test (DART)), those unable to comprehend/communicate, seriously ill/bed ridden patients and those who plan to move out of the study site during the trial period ^39^.
  • *Intervention*: The EHSAS intervention as developed above. The health workers at the villages and the health facilities in the intervention arm will be trained in person on basics of CGA and the intervention, the tools, procedures, and documentation necessary using a standard manual of operating procedures.
  • *Comparator*: Routine practice under the current national programs.
  • *Outcomes*: The primary outcome of interest for the trial is a change in quality-of-life scores (WHO-BREF-Odia version) between baseline and at 12, 18 and 24 months ^40– 43^. Secondary outcomes such as self-rated health, frequency of hospitalization, emergency/out-patient visits and compliance with treatments will be assessed ^44– 46^. The outcomes will be evaluated at individual level.
  • *Data collection and management:* Data on outcomes will be collected in 4 rounds (baseline, 12, 18 and 24 months) by field workers of the DISHA cohort who will be trained in the procedures. The data collectors will use structured electronic forms built for the study and will be blinded to the allocation of the clusters/participants.
  • *Sample size calculation*: We are aiming for a sample size of 100 participants from 10 clusters in this trial. We intend to assess feasibility and power estimations for a future phase-III trial. This sample size is sufficient for an exploratory trial ^47^.
  • *Statistical methods and evaluation of pilot*: We will follow the framework for process evaluation of cluster trials of complex interventions by Grant *et al*. ^48^. Apart from collecting preliminary data on the effect size on outcomes, the feasibility of the pilot will be evaluated by estimating rates of non-participation, drop out, non-consent, loss to follow up and evaluating feasibility of delivering the intervention, measurement of outcomes data, recruitment, randomization, and possible contamination. We will adopt a ‘traffic light’ system for evaluating progression to the main trial and classify outcomes into “Red” zone (unacceptable outcome), “Green” zone (acceptable outcome) or “Amber” zone (potentially acceptable outcome) ^47^.
  • *Data monitoring:* The trial will be monitored by the institutional ethical committee and the Data Safety Management Board. External monitoring and audit will be done by experts not involved in the trial conduct and out of the host institution.
  • *Ethical concerns:* Research ethics approval has been obtained from the Institutional Ethical Clearance Committee of ICMR-RMRC Bhubaneswar (reference ICMR-RMRC/IHEC-2022/109, date: 02/05/2022) and State Health Research Ethics Committee. Protocol has been prospectively registered at Clinical Trial Registry of India and all amendments are noted there. Written informed consent will be obtained from all participants by the study investigators. Confidentiality of the participants and their data will be ensured by strong data safety measures.
  • *Registration and reporting:* The trial has been prospectively registered in the Clinical Trial Registry of India (Ref. No. CTRI/2023/07/055661) which is linked with the World Health Organization’s International Clinical Trials Registry Platform. The findings will be reported following the recommendations of the CONSORT statement specific for cluster RCTs ^49^.

ii. Qualitative assessment
  ▪ Data will be collected from participants using IDIs and FGDs (by investigators, until saturation). These would be translated and transcribed, and thematic analysis will be used to identify the main themes for the outcomes stated below:
    - Reasons for nonparticipation of healthcare workers and clients, drop out, non-consent and loss to follow up.
    - Challenges faced by healthcare workers and data collectors at baseline and follow up to identify the support necessary during scale up.
    - Training needs of healthcare workers with regards to the intervention package
    - Delivery of the intervention and ensuring its consistency and fidelity.

We will use the COREQ checklist for reporting of qualitative findings.

### Feasibility of implementation

**Figure 5.**
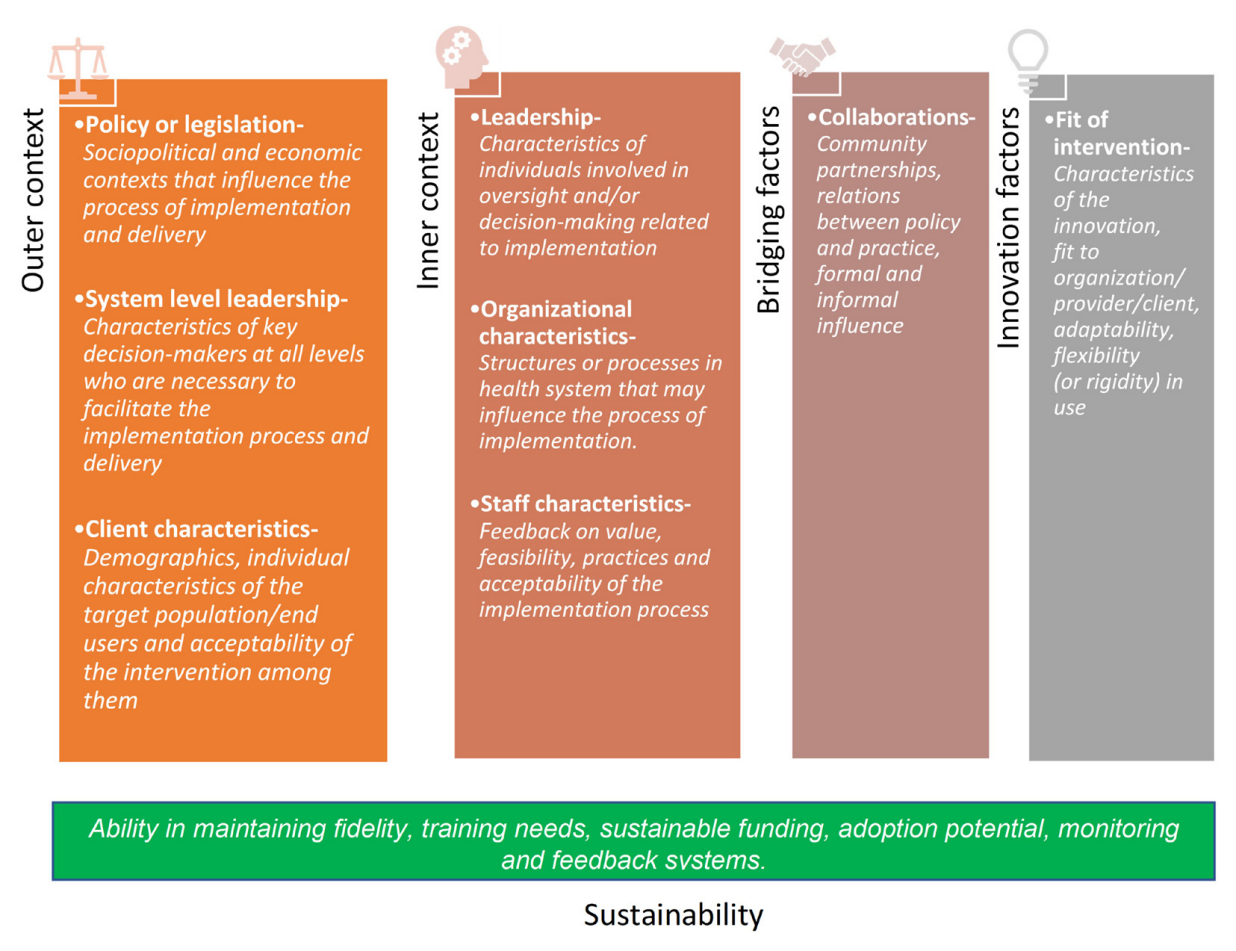
Constructs of EPIS framework adapted for evaluation of implementability of EHSAS.

i. *Pathways*: Feasibility of implementation will be assessed by the following 3 pathways:
  - Identification of current practices related to screening and assessment of older adults in rural areas and the optimization/adaptation/change necessary
  - Barriers and enablers of adoption and implementation
  - Persuasiveness of the evidence needed for adoption and sustainability.

ii. *Methods:* We will use qualitative methods such as FGDs, IDIs, observation checklists and case studies to assess the feasibility of implementation. Participants for this will be selected from the program managers, implementers, heath workers and clients in the study setting and data will be collected by the study investigators.

iii. *Evaluation framework:* Thematic analysis will be done using the selected constructs from the Exploration, Preparation, Implementation and Sustainability (EPIS) framework for evaluation of the implementability of the EHSAS intervention as given below in Figure 5 ^50– 52^.

## Expected outcomes

This study will develop CGA-based interventions, provide the necessary tools and generate evidence on its likely effectiveness and implementability in low- and middle-income settings. CGA based models of care have been recently introduced into the National Programme for Healthcare of the Elderly, and the present study will help build specific tools for use in this ^53^. This will provide a foundation for future phase-III clinical trials and implementation research on the intervention. It will enable me to become a research leader in geriatric health and contribute to service developments for older people in India. This is an exciting opportunity for advancing geriatric public health in India.

## Dissemination

At the start of the project, the study aims will be communicated to the local representatives by attending block level meetings of the elected village officials. Community engagement during the study will be maintained using the existing mechanisms under the platform of the Model Rural Health Research Unit at the study site that includes regular feedback meetings on all projects run by the centre. Similarly, the findings of the study upon completion will be shared with the local representatives, health workers in the block and administrators of the district. We will also make use of social media groups and the website operated by me to disseminate the study findings as well as popular writings such as blogs on health promotion and behavioural issues of the elderly. We will also engage with the health system, policy makers, peers through webinars/seminars, conference papers, scientific publications and policy briefs based on the findings.

## Study status

Participant recruitment will begin from June 2024.

## Data Availability

No underlying data are associated with this article. figshare: Suppl file.dox. https://doi.org/10.6084/m9.figshare.23918490.v1 This project contains the detailed data analysis plan. Data are available under the terms of the Creative Commons Attribution 4.0 International license (CC-BY 4.0). Complete data access will be only available to the core team of the project at ICMR-RMRC. Deidentified data will be shared upon reasonable request for the same from external agencies, in accordance with ICMR data sharing policy. A structured form will be developed for this and an end user licence agreement will be executed with these agencies/individuals. Data security will be ensured by using dedicated in-house servers located at the National Data Centre of IT ministry, Govt. of India, which handles these servers for the host institute. Access to this data will be controlled and a log of the same will be maintained and audited regularly. Direct access will not be available outside India. Data confidentiality will be ensured rigorously by deidentifying the data at the earliest chance. Data that will be shared with external agencies/individuals, or metadata used for interim analysis, or final analysis, will not consist of any participant identifiers. Approval for the data management policy will be sought from the institutional ethical committee of ICMR-RMRC. The findings of the study will be reported following the recommendations of the CONSORT statement specific for cluster RCTs.
