# Supplementary material for: Improving health outcomes among older adults in India: effectiveness and implementability of a novel comprehensive geriatric assessment based intervention
Source: Wellcome Open Res. 2024 Nov 8;8:414. Originally published 2023 Sep 19. [Version 3] doi: 10.12688/wellcomeopenres.19796.3 (PMC11564926; doi:10.12688/wellcomeopenres.19796.3)
Supplement: Peer review history

# Reviewer report: Reviewer response for version 3

**DOI:** 10.21956/wellcomeopenres.25795.r114044

**Published:** 2024-12-13

**Authors:** Chris Gilleard

**Affiliations:** 1 University College London, London, England, UK

While the intent behind the research is inherently worthy, I confess to having a number of reservations. At the same time, having only the most distant knowledge of India’s rural health and social care services, I am hesitant in offering many of my comments as they may well reflect that ignorance. However there are some points that are less contingent upon any local knowledge. My review consequently focuses upon three considerations, based upon my generally supporting in full the study’s overall aim,  of improving the health and wellbeing of older people in rural India.

 In the first place I feel there is undue emphasis upon the techniques of conducting a Comprehensive Geriatric Assessment (CGA) and the importance of ‘validation’ of such CGAs in ‘middle income’ regions of the world.  An outline of CGA in primary care (that is non-institutional based health care) prepared some time ago by the British Geriatric Society  [BGS] focuses clearly upon what a CGA should aim to be -  “a multidimensional holistic assessment of an older person’s health and wellbeing [which] leads to the formulation of a plan to address issues which are of concern to the older person (and their family and carers when relevant)” (BGS, 2019: 3).  Such an assessment is meant to cover signs of a. physical assessment b. functional, social and environmental assessment c. psychological complaints/issues and d. medication review. Arguably these domains are as applicable to rich and poor people and populations. 

 The need is not so much for ‘new tools’ [p.4] but for an  appropriate selection of widely accepted measures, capable of being provided by a nurse, health visitor or similarly qualified paramedic, including    (a) measures of gait and mobility, tests of orientation (time place person), measures of liver and kidney function, blood sugars and lying and standing blood pressure (b) the Barthel or similar measures of functional capacity (c) brief measures of depression (such as the SLU AM SAD 5 item scale) and cognition (such as the Montreal Cognitive Assessment scale) and (d) a review of current medication.  While no ‘single tool’ is ever likely to measure “all the dimensions of CGA” (p.4) such an ‘ideal’ is implausible and for assessment to be ‘comprehensive’ it requires multiple sources of evidence, as the BGS paper explains. 

 My second concern is with the rationale behind effecting (and needing) a distinction between a ‘community based’ and a ‘primary health care’ facility in conducting the assessment. If the aim is to recruit village health workers why not train them to conduct the assessments, and create an MDT format to formulate a care plan which might include clinical or hospital appointments or agree ‘no healthcare intervention’ is needed (or ‘watchful waiting’ only?   This could itself be subject to early trials to see if it is possible (without the time and cost of a two stage process of assessment. The care plan would then frame the basis for evaluating outcomes – how far it was enacted and how far its enactment was successful. Generic outcome data – hospitalisations, clinic visits and frailty outcomes  - seem appropriate (though the mention of ‘frailty index scores’ seems to come out of the blue on p.7).

 My final concern is with the question of sustainability (p.9). It seems to me desirable for there to be a consultation team available throughout that includes health care practitioners, managers, community leaders and members of the local healthcare community (local GPs, nurses and related personnel, including if possible local geriatricians) to provide support, suggestions and when needed questions and criticisms throughout the project.  Such critical friends would I am sure help guarantee some degree of sustainability for what works and what is still needed to effect comprehensive healthcare for older people in rural India.

**References**

 British Geriatric Society (2019) *Comprehensive Geriatric Assessment Toolkit for Primary Care Practitioners*. BGS, London.

 Nasreddine ZS, Phillips NA, Bedirian V, Charbonneau S, Whitehead V, Collin I, Cummings JL, Chertkow H (2005) The Montreal Cognitive Assessment, MoCA: A brief screening tool for mild cognitive impairment. *J Am Geriatr Soc* 53, 695-699.

Is the study design appropriate for the research question?

Yes

Is the rationale for, and objectives of, the study clearly described?

Yes

Are sufficient details of the methods provided to allow replication by others?

Yes

Are the datasets clearly presented in a useable and accessible format?

Yes

Reviewer Expertise:

Ageing, Health & Society

I confirm that I have read this submission and believe that I have an appropriate level of expertise to confirm that it is of an acceptable scientific standard, however I have significant reservations, as outlined above.

# Reviewer report: Reviewer response for version 3

**DOI:** 10.21956/wellcomeopenres.25795.r110697

**Published:** 2024-11-14

**Authors:** Robert Briggs

**Affiliations:** 1 Trinity College Dublin, Dublin, Ireland

Thank you. The authors have adequately addressed my queries / suggestions.

Is the study design appropriate for the research question?

Yes

Is the rationale for, and objectives of, the study clearly described?

Yes

Are sufficient details of the methods provided to allow replication by others?

No

Are the datasets clearly presented in a useable and accessible format?

Not applicable

Reviewer Expertise:

Geriatric Medicine

I confirm that I have read this submission and believe that I have an appropriate level of expertise to confirm that it is of an acceptable scientific standard.

# Reviewer report: Reviewer response for version 2

**DOI:** 10.21956/wellcomeopenres.22945.r106720

**Published:** 2024-11-07

**Authors:** Robert Briggs

**Affiliations:** 1 Trinity College Dublin, Dublin, Ireland

Thank you for the opportunity to read this interesting protocol for the development and pilot of an integrated intervention for older adults in India. The authors outline how they will develop the tool and then test feasibility/efficacy in a cluster RCT.

 I would make the following suggestions:

 1. I think it needs to be made clearer (and this is most likely related to the fact that the tool has not been developed) that the proposed intervention will include a screening tool and then a care plan (rather than screening alone) and what this care plan might look like (allowing for the fact the tool is not yet fully developed). 

 2. I think the authors need to be more precise on what their outcomes will be. It is not currently clear how self-rated health, healthcare utilization and compliance will be assessed. Self-rated health is likely to track very closely to the primary outcome of health-related quality of life. Is there room for objective measures such as mobility? Or other self-reported outcomes like falls? The authors discuss the AHSETS study which collected data on multimorbidity, disability, frailty etc, could this be used to inform the outcomes/data collection in the pilot study?

 3. I think the plain language summary could be revised to increase accessibility. For example the use of terminology like 'primary healthcare facilities' and 'demonstrating feasibility'.

 4. Does Study Status need to be reviewed as it states recruitment will begin in June 2024?

Is the study design appropriate for the research question?

Yes

Is the rationale for, and objectives of, the study clearly described?

Yes

Are sufficient details of the methods provided to allow replication by others?

No

Are the datasets clearly presented in a useable and accessible format?

Not applicable

Reviewer Expertise:

Geriatric Medicine

I confirm that I have read this submission and believe that I have an appropriate level of expertise to confirm that it is of an acceptable scientific standard, however I have significant reservations, as outlined above.

# Response

**Published:** 2024-11-07

**Authors:** Jaya Kshatri

**Affiliations:** Regional Medical Research Centre, Indian Council of Medical Research, Bhubaneswar, Odisha, India

I would like to thank the reviewer for the time and effort put into peer review, and making useful suggestions. Please find our response below:

 1. We agree with this suggestion regarding clearly outlining the intervention , spcifically the use of a novel screeing tool that is being developed, and have made changes in the relevant section of the methodology to reflect this.

 2. We have now expanded more clearly on the outcome measures we will be using in the trial. This has been informed by the AHSETS study outcomes. However, it is important to also note that this being apilot study, we will be focussing more on the feasibility related outcomes, rather than hard efficay end points. The suggestions on including mobility and other outcomes is well taken. We have made changes to the revised version. 

 3. The plain language summary has been edited as advised.

 4. We have revised the study timelines to reflect the current situation. Thank you for pointing this out.

# Reviewer report: Reviewer response for version 1

**DOI:** 10.21956/wellcomeopenres.21927.r67428

**Published:** 2023-11-30

**Authors:** Manasee Panda

**Affiliations:** 1 Department of Community Medicine, Bhima Bhoi Medical College, Balangir, Odisha, India

Authors have mentioned that after a systematic scoping review they could not find any multi-dimensional tools that have been validated yet in South Asia, which can be used in such an intervention.

 This study aims to design and pilot an integrated (Community + Facility) Elderly Health Status Assessment and Screening (EHSAS) intervention to improve health outcomes of older adults and assess its feasibility for implementation in Indian rural settings. For that, the Authors have proposed to develop and validate the Elderly Health Status Assessment and Screening (EHSAS) intervention for community based screening and primary health care based CGA in geriatric population. Then they have planned to test the feasibility and effectiveness of this intervention by a phase-II exploratory cluster randomized controlled trial (RCT and to assess the acceptability of the intervention by the stakeholders. 

1. Already in the year 2021 one study is available on CGA screening ^1^ - a hospital based study which includes 10 domains for screening.
2. Already comprehensive geriatric assessment booklet is available to be used for screening purpose in Geriatric population in community. Already t raining is being imparted to CHOs so that the screening tool can be used. Hence the authors could have mentioned about this in the manuscript and compared the tool already used in the National Programme or mentioned about the challenges in using the tool or about the limitation of the tools. 

Is the study design appropriate for the research question?

Yes

Is the rationale for, and objectives of, the study clearly described?

Yes

Are sufficient details of the methods provided to allow replication by others?

Yes

Are the datasets clearly presented in a useable and accessible format?

Yes

Reviewer Expertise:

As faculty of Community Medicine my areas of research included Medical Education, Maternal & Child Health, Evaluation of Health Programmes.

I confirm that I have read this submission and believe that I have an appropriate level of expertise to confirm that it is of an acceptable scientific standard.

## References

[1] : Comprehensive Geriatric Assessment (CGA) Screening of Geriatric Patients in a Tertiary Care Hospital: a Cross-sectional Study in India. *J Assoc Physicians India* .2022;70(1) :11-12 35062805
